# Retraction Note: Up-regulation of CDK16 by multiple mechanisms in hepatocellular carcinoma promotes tumor progression

**DOI:** 10.1186/s13046-019-1224-x

**Published:** 2019-05-23

**Authors:** Yitao Wang, Xian Qin, Tao Guo, Pengpeng Liu, Ping Wu, Zhisu Liu

**Affiliations:** Department of General Surgery, Research Center of Digestive Diseases, Zhongnan Hospital of Wuhan University, Wuhan, 430071 China

**Retraction Note: J Exp Clin Cancer Res (2017) 36: 97**

**https://doi.org/10.1186/s13046-017-0569-2**

The Editor-in-Chief has retracted this article [1] due to concerns about the figures. In Figure 3C, the images from the colony formation assays of all cell lines, excluding the control for the HCCLM9 cell line, contain identical patterns of colonies; this suggests that despite the fact they are supposed to be observations of different cell lines and different siRNA knockdowns, the images actually depict different sections of the same colony formation assay. This has been confirmed by checking the raw images. For Figure 4D, it has been noticed that the Western blots showing the expression level of E-cadherin are identical, even though they are supposed to be blots from different cell lines. Lastly, for the immunostaining images of CDK16 shown in Figure 7C, it is evident from the nuclei size in the two images that different zoom-factors have been used, yet the scale-bars indicate 50μm for both images. Due to these errors, the Editor-in-Chief has concerns about the reliability of the results presented in the article. Yitao Wang, Xian Qin and Zhisu Liu agree to this retraction. Tao Guo, Pengpeng Liu and Ping Wu have not responded to any correspondence from the publisher about this retraction.
